# Clarifying the association of CSF Aβ, tau, BACE1, and neurogranin with AT(N) stages in Alzheimer disease

**DOI:** 10.1186/s13024-024-00755-3

**Published:** 2024-10-08

**Authors:** Sylvain Lehmann, Susanna Schraen-Maschke, Luc Buée, Jean-Sébastien Vidal, Constance Delaby, Christophe Hirtz, Frédéric Blanc, Claire Paquet, Bernadette Allinquant, Stéphanie Bombois, Audrey Gabelle, Olivier Hanon, for the Alzheimer’s Disease Neuroimaging Initiative (ADNI)

**Affiliations:** 1 LBPC-PPC, Université de Montpellier, INM INSERM, IRMB CHU de Montpellier, 80 av Fliche, Montpellier, 34295 France; 2 Univ. Lille, Inserm, CHU Lille, UMR-S-U1172, LiCEND, Lille Neuroscience & Cognition, LabEx DISTALZ, Lille, F-59000 France; 3 Université Paris Cité, EA 4468, APHP, Hospital Broca, Memory Resource and Research Centre of de Paris-Broca-Ile de France, Paris, F-75013 France; 4 Sant Pau Memory Unit, Hospital de la Santa Creu i Sant Pau - Biomedical Research Institute Sant Pau - Universitat Autònoma de Barcelona, Barcelona, Spain; 5 Université de Strasbourg, CHRU de Strasbourg, Memory Resource and Research Centre of Strasbourg/Colmar, French National Centre for Scientific Research (CNRS), ICube Laboratory and Fédération de Médecine Translationnelle de Strasbourg (FMTS), Team Imagerie Multimodale Intégrative en Santé (IMIS)/Neurocrypto, Strasbourg, F-67000 France; 6 Université Paris Cité, GHU APHP Nord Lariboisière Fernand Widal, Centre de Neurologie Cognitive, Paris, F-75010 France; 7 UMR-S1266, Université Paris Cité, Institute of Psychiatry and Neuroscience, Inserm, Paris, France; 8 Assistance Publique-Hôpitaux de Paris (AP-HP), Département de Neurologie, Centre des Maladies Cognitives et Comportementales, GH Pitié-Salpêtrière, Paris, France; 9 Université de Montpellier, Memory Research and Resources Center, Department of Neurology, Inserm INM NeuroPEPs team, Montpellier, F-34000 France

**Keywords:** Alzheimer’s disease, Amyloid, BACE1, Cerebrospinal fluid, Neurodegeneration, Neurogranin, Tau proteinopathy

## Abstract

**Background:**

Current AT(N) stratification for Alzheimer’s disease (AD) accounts for complex combinations of amyloid (A), tau proteinopathy (T) and neurodegeneration (N) signatures. Understanding the transition between these different stages is a major challenge, especially in view of the recent development of disease modifying therapy.

**Methods:**

This is an observational study, CSF levels of Tau, pTau181, pTau217, Aβ38/40/42, sAPPα/β, BACE1 and neurogranin were measured in the BALTAZAR cohort of cognitively impaired patients and in the Alzheimer's Disease Neuroimaging Initiative (ADNI). Biomarkers levels were related to the AT(N) framework. (A) and (T) were defined in BALTAZAR with CSF Aβ42/40 ratio and pTau217 respectively, and in ADNI with amyloid and tau PET. (N) was defined using total CSF tau in both cohorts.

**Results:**

As expected, CSF Aβ42 decreased progressively with the AD continuum going from the A-T-N- to the A + T + N + profile. On the other hand, Tau and pTau181 increased progressively with the disease. The final transition from A + T + N- to A + T + N + led to a sharp increase in Aβ38, Aβ42 and sAPP levels. Synaptic CSF biomarkers BACE1 and neurogranin, were lowest in the initial A + T-N- stage and increased with T + and N + . CSF pTau181 and total tau were closely related in both cohorts.

**Conclusions:**

The early transition to an A + phenotype (A + T-N-) primarily impacts synaptic function. The appearance of T + and then N + is associated with a significant and progressive increase in pathological Alzheimer's disease biomarkers. Our main finding is that CSF pTau181 is an indicator of N + rather than T + , and that N + is associated with elevated levels of BACE1 protein and beta-amyloid peptides. This increase may potentially fuel the amyloid cascade in a positive feedback loop. Overall, our data provide further insights into understanding the interconnected pathological processes of amyloid, tau, and neurodegeneration underlying Alzheimer's disease.

**Supplementary Information:**

The online version contains supplementary material available at 10.1186/s13024-024-00755-3.

## Background

The [AT(N)] classification was proposed in 2018 by the National Institute on Aging and Alzheimer’s Association (NIA-AA). The system uses longitudinal imaging and biomarker studies of patients with cognitive decline or at risk of developing Alzheimer’s disease (AD) [1]. This classification refers primarily to the presence of amyloid (A) and tau (T) pathology, which are the hallmarks of AD. The presence of neurodegeneration (N) was also considered a marker for advanced pathologic state that could be employed for staging. ‘N’ was put into parenthesis because neurodegeneration does “not map onto neuropathologic findings used to diagnose AD” [1]. Recent revision of the NIA-AA clinical guidelines proposed retaining just ‘A’ and ‘T’ proteinopathy status for diagnosis and staging of AD. ‘N’ thus became a second-tier marker, along with ‘I’ (inflammation) and co-pathologies. The AT(N) classification is a very constructive framework to model the sequential pathological events in AD and to dissect out biomarkers related to A, T and N.

The interaction between amyloid (A) and tau (T) pathology, and the initiating role of either one or the other, have been much debated. The amyloid hypothesis [2] proposes that AD initiates through the accumulation of amyloid-beta (Aβ) peptides in the brain. Aβ peptides are generated from the amyloid precursor protein (APP) and they build up in the brain due to an imbalance between production and clearance [3]. Many factors could be responsible for an increase in Aβ metabolic dysregulation; first and foremost are mutations in the APP and γ-secretase genes. Indeed APP duplication and trisomy 21 both cause early-onset AD [4]. Dysregulation of β-site APP cleaving enzyme 1 (BACE1) by inflammatory factors, oxidative stress [5], hormone signalling (e.g. insulin), mitochondrial dysfunction and altered lipid metabolism, can also increase Aβ production [6]. Otherwise, impaired degradation of Aβ might result from lower activity of enzymes, like neprilysin and insulin-degrading enzyme, or from impaired autophagy or proteasome activity [7].

Microglial activity also impacts amyloid deposition. Indeed, several genetic AD-risk factors are linked to microglial cell fate [8]. Clearance of Aβ peptides from the brain is affected by dysfunction in blood–brain barrier or glymphatic system [9]. Aging, but also sleep disruption [10], brain injury and trauma and other environmental factors can also influence these systems to reduce Aβ peptide clearance and thus increase AD risk.

It is still a matter of debate whether amyloid plaques or tau tangles are the dominant pathology in AD. Certainly, the earliest pathological biomarkers detected in the AD continuum appear to be amyloid [11]. It has also been established that anti-Aβ immunotherapies reduce biomarkers associated with both amyloid and tau tangle pathology [12]. In this context, amyloid represents a trigger for tau pathology [12–14]. Nevertheless, tau pathology can also occur without amyloid, as reported in cases of “suspected non-Alzheimer's disease pathophysiology” (SNAP) [1, 15, 16] and “primary age-related tauopathy” (PART) [17]. Tau pathology is thus a parallel path to dementia that is closely linked to the classical AD symptoms of cognitive decline, neurodegeneration and synaptic dysfunction. The progress of these physiological parameters in AD can, in turn, be monitored by CSF levels of neurogranin (Ng) and base BACE1 or the ratio between the two [18]. The classic AD continuum can thus be defined as the transition from a healthy state (A-T-) to cerebral amyloidosis (A + T-), then to patients with a full AD profile (A + T +) [19], with isolated tau proteinopathy (A-T +) completing the spectrum. Understanding, predicting and controlling the transition between these different states is a major challenge, especially in view of the recent development of disease modifying therapy.

CSF biomarkers can provide clues to AD pathophysiology and many studies focus on the Aβ42 peptide or the Aβ42/40 ratio [20]. However, while these measurements can indeed be used to monitor A + , they do not provide an adequate picture of Aβ metabolism within the AD continuum. Here we carefully monitor non-amyloidogenic Aβ38 or Aβ40 peptides [21] along with associated sAPP fragments. In addition to amyloid and tau CSF biomarkers, we also measured levels of Ng and BACE1, as both have been proposed as synaptic biomarkers relevant for AD cognitive decline [18, 22, 23]. As an enzyme, BACE1 cleaves the N-terminus of APP at different moieties, generating at least three different Aβ peptides after γ-secretase action: Aβ38, Aβ40 and Aβ42, and the corresponding soluble fragment sAPPβ [24]. We included all these biomarkers in our study, as well sAPPα which is link to the non-amyloidogenic metabolism of APP. These species are all known to correlate with one another in physiological situations [21]. We observed that isolated A + has mainly a synaptic impact, then, combined with T + and N + , it contributes to the classic AD profile. In this progression, N + is associated with a significant increase in pathological biomarkers but is also characterized by surprisingly high concentrations of Aß40 peptides and BACE1. This increase potentially fuelling the amyloid cascade, provides further clues to understanding the link between the amyloid and tau pathological processes underlying AD.

## Methods

### Baltazar study population

The study population corresponds to 209 participants of the BALTAZAR multicenter prospective cohort (ClinicalTrials.gov Identifier #NCT01315639) [25] who underwent lumbar puncture as part of the clinical protocol. All participants had clinical, neuropsychological, imaging and biological assessments. APOE was genotyped in a single centralized laboratory. Mild cognitive impairment subjects (MCI) were selected according to the Petersen’ criteria [26]. Participants had visits every six months for three years, where they were reassessed each time for cognitive decline [25].

### Biological biomarker measurements in the Baltazar cohort

CSF samples were taken at the first visit, and to minimize pre-analytical and analytical problems, identical collection tubes were used across centers. CSF aliquots were stored at -80 °C in low-binding Eppendorf® LoBind microtubes (Eppendorf, ref 022431064, Hamburg, Germany) until testing. Biomarker levels, of Tau, pTau181, Aβ40 and Aβ42, were determined with standardized commercially available ELISA Kits (Euroimmun β-amyloid 1–40 and 1–42 [27], Innotest hTau [28], and Innotest Phospho-Tau (181P) [29]). CSF pTau217 was determine using the commercial MSD (Meso Scale Discovery, Rockville, MD, USA) S-PLEX Human Tau (pT217) Kit. CSF sAPPα, sAPPβ and Aβ38 were detected using multiplex MSD kits (ref: K11120E, K11148E). Detailed assay procedures, that were very similar to classical ELISA, but with a final quantitation on the MSD Sector Imager 6000 plate reader, are provided elsewhere [21]. The immunoassay detecting protein levels of BACE1 is an ELISA further developed from a format described by Barao and colleagues [30], including monoclonal antibodies ADx401 (clone 5G7) and ADx402 (clone 10B8F1). BACE1 levels were measured according to the kit instructions, where concentrations were calculated via intrapolation (5PL curve fit; log (X)) based on the calibrator curve. To measure Ng, an adapted version of the originally described format [18] was used. In short, this assay includes two monoclonal antibodies, ADx403 (clone ADxNGCI2) and ADx451 (clone ADxNGCT1), that specifically capture neurogranin C-terminally truncated at proline 75 (P75), which is abundant in CSF [31]. BACE1 and Ng ELISA are commercialized by Euroimmun and include ready-to-use, lyophilized calibrators and a standardized protocol.

### ADNI study population

Two sets of data, originating from the Alzheimer's Disease Neuroimaging Initiative (ADNI) (www.loni.ucla.edu/ADNI), were used after the agreement of the scientific committee. The first dataset ADNI 1, was generated using data from UPENNBIOMK_MASTER_FINAL that contains CSF data (AB40, ABETA, PTAU, TAU) measured with the Elecsys® platform combined with Aβ PET (UCBERKELEY_AMY_6MM) and tau PET data (UCBERKELEY_TAUPVC_6MM). We retained 512 CSF samples with Aβ and tau PET status determined within 4 months of lumbar puncture (mean delay between lumbar puncture and PET was 16.6(± 22.4) days for Aβ PET and 20.7(± 28.4) days for tau PET). An ADNI subset is represented by the “Biomarkers Consortium Project BACE activity and sAPPβ measures as Novel Cerebrospinal Fluid” (*n* = 377). In this cohort, CSF BACE1 activity and sAPPβ were measured using validated methods described elsewhere [32]. Concentrations of amyloid and tau biomarker (ABETA, PTAU, TAU), measured with the Elecsys® platform were also retrieve from the UPENNBIOMK_MASTER_FINAL.

### Stratification of the ADNI and BALTAZAR cohorts based on AT(N) status

In the ADNI cohort, we relied on PET analysis to determine the amyloid Aβ (A +) and tau (T +) status. The cutpoints for Aβ PET positive status (FBP: 1.11/20 CL, FBB: 1.08/18 CL) and for tau status (1.26; temporal meta SUVr) are described in Landau et al. [33]. In the Baltazar cohort, A + status was based on well-established cutpoint for the CSF Aβ42/40 ratio (i.e. < 0.10) [27]. The Aβ42/40 ratio is known to robustly predict Aβ PET status [34]. In previous work from our teams [35, 36], as well as from others [37–39], pTau181 was used to define T + status with a cutpoint of 60 ng/mL. However, since in the BALTAZAR cohort we also measured CSF pTau217, which performs significantly better than pTau181 for diagnosis [40] and to predict tau PET status [41], we thus relied on pTau217 to determine (T) status with a cutpoint of 242 ng/mL. Neurodegenerative status (N) is commonly based on the value of the total Tau level in the CSF. We therefore used previously defined Tau cut-points of 400 pg/mL using the corresponding immunoassays [27, 42], in both cohorts.

### Statistical analyses

General characteristics were analysed in the MCI Baltazar populations with different ATN profiles. Categorical variables were analysed as percentage (%), and continuous variables as mean and standard deviation (M (SD)) or as median (25–75 percentile) after testing for normal distribution by Shapiro–Wilk test. Comparisons were then made by χ^2^ test, T-test, or Wilcoxson test. Differences in Kaplan–Meier biomarkers tertiles were calculated by Log rank test. The focus of the study is not on ApoeE4, and to avoid biases linked to this variable as well as to age and sex, statistical comparisons were adjusted with these three factors. For all analyses, a 2-sided α-level of 0.05 was used for significance testing. All analyses were performed using MedCalc (20·118) and R (Core Team 2019) software.

## Results

### Demographics and CSF biomarkers along the AD continuum: A-T-N-, A + T-N-, A + T + N- to A + T + N + 

**Fig. 1:**
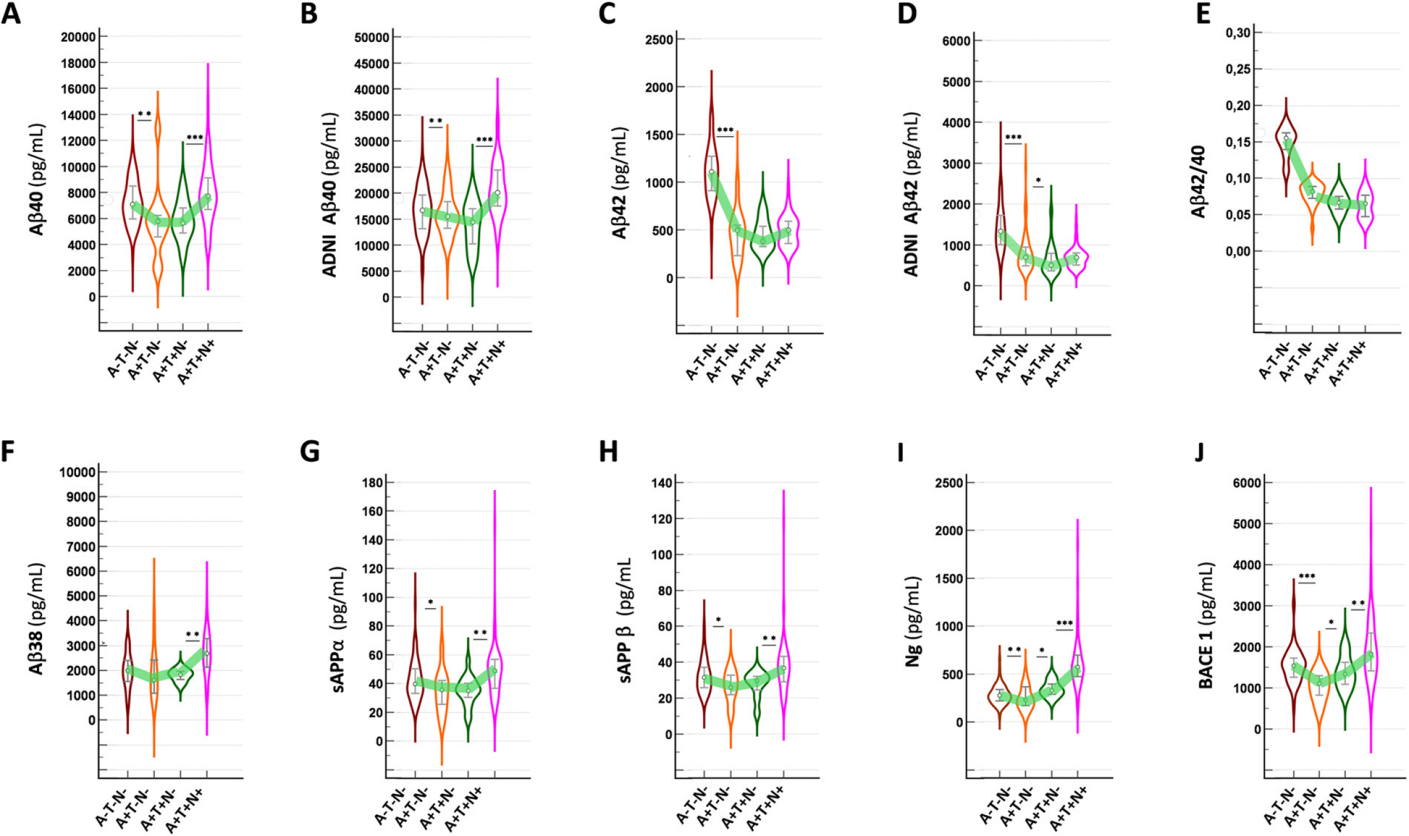
CSF biomarker levels in the AT(N) framework. Violin plot distribution of Aβ40 and Aβ42 CSF levels in the BALTAZAR (**A**, **C**) and the ADNI (**B**, **D**) cohorts, stratified by AT(N) classification showing median and quartiles. Aβ40 levels were statistically different between A-T-N- vs. A + T-N- and between A + T + N- vs. A + T + N + . Aβ38 levels (**F**) showed also an statistically significant increase with the presence of N + . Aβ42 levels as well as Aβ42/40 ratio (**E**) is used for A + stratification were much lower in A + T-N- compared to A-T-N-. sAPPs distribution (**G**, **H**) are similar to that of Aβ40. Ng (**I**), as a synaptic biomarker, is decreased in isolated A + T-N- and increased in T + and N + . BACE1 (**H**) is also decreased in isolated A + T-N-, increased a little in T + and more in N + . *P* values of Wilcoxson test < 0.001 are indicated with ***, < 0.01 with ** and < 0.05, with *

In the 209 MCI participants of the BALTAZAR cohort who had CSF analysis, 32.1% were A-T-N, 12.4% A + T-N-, 1.9% A + T + N- and 36.4% A + T + N + (Supplementary Tables 1 and 2, Supplementary Fig. 1). In the ADNI cohort, which assembles AD and non-AD patients, these numbers were 41.0%, 11.5%, 10.5% and 18.9%, respectively. The BATAZAR cohort shows higher values in the different ATN groups, as it represents MCI patients which are older and overall more advanced along the AD continuum than ADNI patients. These numbers differ significantly from the stratification of the cohorts based on the presence of one the AT(N) hallmarks (Supplementary Tables 1 and 2). In both cohorts (Table 1), pathological subsets were slightly older than non-pathological A-T-N- participants, with a higher percentage of ApoE4 carriers, faster cognitive decline, and a gender-equivalent distribution. We focused on group comparison based on the AD continuum: i.e. the acquisition of A + (A-T-N- vs A + T-N-), T + (A + T-N- vs A + T + N-) and N + (A + T + N- vs A + T + N +) statuses. The appearance of A + , determined using CSF Aβ42/40 in BALTAZAR and amyloid PET in ADNI, was associated with an over-representation of ApoE4, cognitive decline, Aβ42, BACE1 and Ng decrease, and an increase of Tau, pTau181 and pTau217 (Table 1, Fig. 1). The arrival of T + , determined using CSF pTau217 in BALTAZAR and tau PET in ADNI, was only associated with a small decrease of Aβ42, Aβ42/40 and a small increase of BACE1 and pTau181. Hippocampal volume was significantly decreased in the ADNI cohort, which includes both AD and non-AD patients. Major changes were observed with the addition of N + , determined using CSF Tau, with a further rise of all AD biomarkers, and a significant increase in BACE1 and metabolic amyloid biomarkers including Aβ38, Aβ40, sAPPα and sAPPβ (Table 1, Fig. 1). It should be noted that stratifying cohorts according to the presence or absence of one of the AT(N) features gives a general and less accurate picture of the impact of the AT(N) component on CSF biomarkers (Supplementary Table 3, Supplementary Fig. 2).

**Table 1:** Demographics and CSF biomarker values in the number (n) of participants in within the AD continuum (A-T-N-, A + T-N-, A + T + N- and A + T + N +). Comparison between A-T-N- vs A + T-N-, A + T-N- vs A + T + N- and A + T + N- vs A + T + N + profiles. Categorical variables were reported as percentage (%), continuous variables mean (standard deviation) or as median (25-75 percentile) after testing for normal distribution using the Shapiro-Wilk test. This explains why, for the same subgroup of patients, a variable could be represented either as the mean (standard deviation), italicized, or as the median (25th-75th percentile). *P* values of comparison made by χ^2^ test, T-test, or Wilcoxson test. *P* $ values of comparison with linear regression adjusted for age, sex, and the presence of the APOE ε4 allele. *** indicates the biomarker used for stratification

**Baltazar cohot**	**n**	**A-T-N-**	**n**	**A+T-N-**	**P**	**P$**	**n**	**A+T-N-**	**n**	**A+T+N-**	**P**	**P$**	**n**	**A+T+N-**	**n**	**A+T+N+**	**P**	**P$**	**Variable**
Age (year)	71	*76.5 (4.9)*	11	*79.9 (6.4)*	0.115	/	11	*79.9 (6.4)*	19	*78.9 (5.0)*	0.6737	/	19	*78.9 (5.0)*	81	*77.5 (6.0)*	0.282	/	Age (year)
Men/Women	71	42.3%	11	36.4%	0.968	/	11	36.4%	19	31.6%	1	/	19	31.6%	81	38.3%	0.7796	/	Men/Women
e4/total genotyped	71	11.3%	11	36.4%	0.0831	/	11	36.4%	19	63.2%	0.2993	/	19	63.2%	81	55.6%	0.7301	/	e4/total genotyped
Hippocampal vol	60	*4.72 (1.21)*	8	*5.02 (0.68)*	0.3101	0.1166	8	*5.02 (0.68)*	17	*4.40 (0.83)*	0.0614	0.4137	17	*4.40 (0.83)*	70	*4.45 (0.97)*	0.8152	0.961	Hippocampal volume (R+L) (cm3)
MMSE	70	28.0 (26.0-29.0)	10	26.0 (25.2-27.0)	0.0595	0.0533	10	*25.7 (2.5)*	18	*25.6 (3.1)*	0.8943	0.8046	18	25.5 (23.2-28.5)	80	26.0 (24.0-28.0)	0.8097	0.5497	MMSE
MMSE/year	66	-0.33 (-1.18-0.07)	10	-0.98 (-2.20-0.14)	0.6952	0.6271	10	-0.98 (-2.20-0.14)	18	-0.19 (-1.26-0.20)	0.5814	0.8768	18	-0.19 (-1.26-0.20)	72	-1.06 (-4.10--0.01)	0.0892	0.4497	MMSE/year
Aβ38 (pg/mL)	66	*2051 (795)*	11	*1980 (1165)*	0.8493	0.3544	11	*1980 (1165)*	17	*1789 (764)*	0.6369	0.8092	17	*1789 (764)*	75	*2559 (844)*	0.0011	0.0008	Aβ38 (pg/mL)
Aβ40 (pg/mL)	71	7086 (5992-8448)	11	5791 (4626-6204)	0.0077	0.0031	11	5791 (4626-6204)	19	5678 (4946-6754)	0.6359	0.8359	19	*5845 (1532)*	81	*7965 (2285)*	< 0.0001	0.0002	Aβ40 (pg/mL)
Aβ42 (pg/mL)	71	1105 (275)	11	457 (236)	< 0.0001	< 0.0001	11	*457 (236)*	19	*449 (158)*	0.9203	0.8386	19	382 (324-530)	81	494 (357-589)	0.3144	0.3427	Aβ42 (pg/mL)
Aβ42/40	71	0.156 (0.145-0.164)	11	0.086 (0.078-0.089)	***	***	11	0.086 (0.078-0.089)	19	0.076 (0.067-0.088)	0.3223	0.9236	19	0.076 (0.067-0.088)	81	0.065 (0.048-0.076)	0.0029	0.0006	Aβ42/40
sAPPα (pg/mL)	66	31.4 (25.7-36.9)	11	26.0 (22.4-32.2)	0.103	0.0094	11	26.0 (22.4-32.2)	17	29.7 (25.8-31.9)	0.7778	0.8902	17	29.7 (25.8-31.9)	75	36.5 (29.1-43.0)	0.0015	0.0058	sAPPα (pg/mL)
sAPPβ (pg/mL)	66	39.8 (33.3-50.2)	11	35.7 (25.8-41.9)	0.1228	0.0175	11	*34.8 (14.7)*	17	*34.9 (10.1)*	0.9785	0.9316	17	35.2 (32.2-39.4)	75	49.1 (37.0-57.0)	0.0007	0.0021	sAPPβ (pg/mL)
Tau (pg/mL)	71	274 (212-328)	11	219 (170-302)	0.2081	0.0078	11	219 (170-302)	19	335 (280-372)	0.0201	0.0106	19	335 (280-372)	81	627 (542-805)	***	***	Tau (pg/mL)
pTau181 (pg/mL)	71	*46.7 (10.6)*	11	*41.5 (13.2)*	0.2336	0.008	11	*41.5 (13.2)*	19	*51.3 (11.4)*	0.0538	0.0305	19	51.0 (45.5-55.0)	81	88.0 (70.0-111.0)	< 0.0001	< 0.0001	pTau181 (pg/mL)
pTau217 (pg/mL)	71	108.4 (79.8-135.0)	11	187.4 (168.3-195.4)	0.0001	0.0001	11	187.4 (168.3-195.4)	19	351.0 (294.8-479.7)	***	***	19	351.0 (294.8-479.7)	81	619.4 (468.0-967.3)	< 0.0001	0.0019	pTau217 (pg/mL)
BACE1 (pg/mL)	71	1534 (1260-1719)	11	1100 (824-1274)	0.0004	< 0.0001	11	*1022 (353)*	19	*1381 (394)*	0.0169	0.0247	19	1335 (1096-1568)	81	1819 (1420-2336)	0.0018	0.0046	BACE1 (pg/mL)
Ng (pg/mL)	71	281 (221-363)	11	179 (107-264)	0.0097	0.0002	11	*195 (113)*	19	*292 (113)*	0.0352	0.0242	19	256 (213-372)	81	544 (400-687)	< 0.0001	< 0.0001	Ng (pg/mL)
Ng/BACE1	71	*19.8 (6.2)*	11	*18.7 (7.2)*	0.6342	0.3168	11	*18.7 (7.2)*	19	*21.3 (6.6)*	0.3328	0.2631	19	20.5 (17.4-23.7)	81	28.5 (21.7-32.7)	0.0006	0.0133	Ng/BACE1
**ADNI cohort**	**n**	**A-T-N-**	**n**	**A+T-N-**	**P**	**P$**	**n**	**A+T-N-**	**n**	**A+T+N-**	**P**	**P$**	**n**	**A+T+N-**	**n**	**A+T+N+**	**P**	**P$**	**Variable**
Age (year)	210	69.4 (65.6-75.0)	59	73.6 (69.2-78.0)	0.0006	0.0008	59	*73.8 (6.7)*	54	*72.8 (7.4)*	0.5121	0.5998	54	*72.8 (7.4)*	97	*74.1 (7.9)*	0.3769	0.3633	Age (year)
Men/Women	210	47.3%	59	49.1%	0.9401	0.8796	59	49.1%	54	44.4%	0.7935	0.7989	54	44.4%	97	48.00%	0.8501	0.8403	Men/Women
e4/total genotyped	210	19.1%	59	54.5%	< 0.0001	< 0.0001	59	54.5%	54	57.8%	0.9033	0.7935	54	57.8%	97	65.3%	0.5268	0.3902	e4/total genotyped
Hippocampal vol	210	*8035 (927)*	59	*7938 (902)*	0.4718	0.6122	59	*7938 (902)*	54	*7228 (947)*	0.0001	< 0.0001	54	*7228 (947)*	97	*7032 (1140)*	0.2606	0.2519	Hippocampal volume
Aβ40 (pg/mL)	210	*16637 (4568)*	59	*15919 (4254)*	0.2623	0.3533	59	15500 (13320-18230)	54	14490 (10295-16958)	0.0602	0.0276	54	14490 (10295-16958)	97	20120 (17560-24390)	< 0.0001	< 0.0001	Aβ40 (pg/mL)
Aβ42 (pg/mL)	210	1334 (1007-1722)	59	706 (497-947)	< 0.0001	< 0.0001	59	706 (497-947)	54	513 (369-794)	0.0079	0.006	54	513 (369-794)	97	689 (516-803)	0.019	0.0437	Aβ42 (pg/mL)
Aβ42/40	210	0.087 (0.074-0.095)	59	0.047 (0.038-0.056)	< 0.0001	< 0.0001	59	0.047 (0.038-0.056)	54	0.042 (0.033-0.050)	0.0821	0.0137	54	0.042 (0.033-0.050)	97	0.032 (0.029-0.038)	< 0.0001	< 0.0001	Aβ42/40
Tau (pg/mL)	210	186 (154-228)	59	223 (187-246)	0.0004	0.0035	59	223 (187-246)	54	241 (195-257)	0.2144	0.253	54	241 (195-257)	97	432 (341-504)	***	***	Tau (pg/mL)
pTau181 (pg/mL)	210	15.5 (12.4-19.8)	59	19.7 (16.2-22.9)	< 0.0001	0.0001	59	19.7 (16.2-22.9)	54	22.6 (17.7-24.8)	0.0372	0.047	54	22.6 (17.7-24.8)	97	43.0 (33.4-54.8)	< 0.0001	< 0.0001	pTau181 (pg/mL)

Abbreviations: *e4* apolipoprotein E4, Mini–Mental State Examination, *sd* standard deviation, *Ng* neurogranin, *BACE1* β-site APP cleaving enzyme 1

### Demographics and CSF biomarkers in isolated T + (A-T + N-) and N + (A-T-N +) profiles

The number of patients with A-T + N- and A-T-N + profiles, that do not belong to the Alzheimer's disease continuum, is limited (< 5%) (Supplementary Table 1 and Table 2). A-T + N-, defined with tau PET in ADNI or CSF pTau217 in BALTAZAR, was not associated with a significant increase of CSF total tau or pTau181 in either cohort, nor did it have a significant impact on amyloid biomarkers. A-T-N + defined with CSF total tau, is associated with high pTau181 but not with high pTau217. Importantly, A-T-N + is associated with Aβ40 and BACE1 increased levels in both cohorts. This was also observed when global N + population were analysed (Supplementary Tables 3 and 4).

**Table 2:** Comparison of values in the number (n) of participants within the different status. Categorical variables were reported as percentage (%), continuous variables as mean (standard deviation) or as median (25-75 percentile) after testing for normal distribution using the Shapiro-Wilk test. This explains why, for the same subgroup of patients, a variable could be represented either as the mean (standard deviation), italicized, or as the median (25th-75th percentile). *P* values of comparison made by χ^2^ test, T-test, or Wilcoxson test. *P* $ values of comparison with linear regression adjusted for age, sex, and the presence of the APOE ε4 allele. *** indicates the biomarker used for stratification

**Baltazar cohot**	**n**	**A-T-N-**	**n**	**A-T+N-**	**P**	**P$**	**n**	**A-T-N-**	**n**	**A-T-N+**	**P**	**P$**
Age (year)	71	*76.5 (4.9)*	6	*74.0 (3.1)*	0.1146	/	71	*76.5 (4.9)*	11	*77.3 (6.2)*	0.6993	/
Men/Women	71	42.3%	6	33.3%	1.0000	/	71	42.3%	11	63.6%	0.3170	/
e4/total genotyped	71	11.3%	6	50.0%	0.0459	/	71	11.3%	11	27.3%	0.3301	/
Hippocampal vol	60	*4.72 (1.21)*	6	*5.38 (1.01)*	0.1782	0.5884	60	*4.72 (1.21)*	9	*3.74 (1.26)*	0.0527	0.0112
MMSE	70	28.0 (26.0-29.0)	5	28.0 (24.0-29.0)	0.9400	0.7675	70	28.0 (26.0-29.0)	10	26.5 (25.2-27.0)	0.0992	0.3159
MMSE/year	66	-0.33 (-1.18-0.07)	6	-0.14 (-0.48-0.10)	0.3978	0.6189	66	-0.33 (-1.18-0.07)	9	-0.59 (-3.06-0.07)	0.5962	0.4685
Aβ38 (pg/mL)	66	*2051 (795)*	6	*2231 (1026)*	0.6920	0.9859	66	*2051 (795)*	11	*3217 (1290)*	0.0139	0.0003
Aβ40 (pg/mL)	71	*7224 (1677)*	6	*6664 (1682)*	0.4633	0.3548	71	*7224 (1677)*	11	*8908 (2352)*	0.0419	0.0035
Aβ42 (pg/mL)	71	*1105 (275)*	6	*765 (213)*	0.0092	0.0038	71	*1105 (275)*	11	*1236 (173)*	0.0456	0.0583
Aβ42/40	71	0.156 (0.145-0.164)	6	0.110 (0.106-0.112)	0.0007	< 0.0001	71	0.156 (0.145-0.164)	11	0.148 (0.124-0.161)	0.2824	0.3607
sAPPα (pg/mL)	66	31.4 (25.7-36.9)	6	30.8 (24.8-37.6)	0.8705	0.2991	66	31.4 (25.7-36.9)	11	30.8 (28.0-32.4)	0.5030	0.2324
sAPPβ (pg/mL)	66	39.8 (33.3-50.2)	6	40.6 (31.6-49.4)	0.8625	0.2772	66	39.8 (33.3-50.2)	11	37.5 (35.6-45.5)	0.8102	0.5037
Tau (pg/mL)	71	274 (212-328)	6	315 (287-344)	0.0982	0.0547	71	274 (212-328)	11	481 (456-586)	***	***
pTau181 (pg/mL)	71	46.7 (10.6)	6	53.7 (6.7)	0.0532	0.0297	71	*46.7 (10.6)*	11	*72.3 (14.6)*	0.0001	< 0.0001
pTau217 (pg/mL)	71	111.8 (42.0)	6	334.4 (81.9)	***	***	71	*111.8 (42.0)*	11	*147.1 (57.5)*	0.0746	0.0553
BACE1 (pg/mL)	71	1534 (1260-1719)	6	1552 (1239-1710)	0.7829	0.7340	71	1534 (1260-1719)	11	1672 (1498-2560)	0.0413	0.0022
Ng (pg/mL)	71	281 (221-363)	6	316 (255-348)	0.5494	0.9698	71	281 (221-363)	11	357 (306-760)	0.0257	0.0002
Ng/BACE1	71	18.3 (15.6-24.5)	6	21.6 (3.6)	0.2962	0.7860	71	18.3 (15.6-24.5)	11	24.2 (19.3-26.9)	0.0534	0.0480
**ADNI cohort**	**n**	**A-T-N-**	**n**	**A-T+N-**	**P**	**P$**	**n**	**A-T-N-**	**n**	**A-T-N+**	**P**	**P$**
Age (year)	210	69.4 (65.6-75.0)	20	72.3 (68.9-76.8)	0.1257	/	210	69.4 (65.6-75.0)	33	70.1 (67.0-76.1)	0.5047	/
Men/Women	210	47.3%	20	50.0%	1.0000	/	210	47.3%	33	50.0%	0.9410	/
e4/total genotyped	210	19.1%	20	22.2%	0.9976	/	210	19.1%	33	33.3%	0.1267	/
Hippocampal vol	210	*8035 (927)*	20	*7471 (1021)*	0.0263	0.0441	210	*8035 (927)*	32	*8119 (945)*	0.6408	0.9022
Aβ40 (pg/mL)	210	*16637 (4568)*	20	*16387 (5792)*	0.8527	0.7342	210	*16637 (4568)*	33	*26788 (4086)*	< 0.0001	< 0.0001
Aβ42 (pg/mL)	210	1334 (1007-1722)	20	1268 (801-1708)	0.3128	0.8344	210	1334 (1007-1722)	33	2026 (1490-2778)	< 0.0001	< 0.0001
Aβ42/40	210	0.087 (0.074-0.095)	20	0.080 (0.067-0.096)	0.3708	0.6647	210	0.087 (0.074-0.095)	33	0.080 (0.057-0.097)	0.4265	0.8901
Tau (pg/mL)	210	186 (154-228)	20	202 (163-256)	0.3450	0.5344	210	186 (154-228)	33	331 (305-362)	***	***
pTau181 (pg/mL)	210	15.5 (12.4-19.8)	20	16.5 (13.6-22.1)	0.4288	0.6010	210	15.5 (12.4-19.8)	33	28.9 (26.8-30.9)	< 0.0001	< 0.0001
**Variable Baltazar cohot**	**n**	**CSF_pTau181-**	**n**	**CSF_pTau181+**	**P**	**P$**	**n**	**A-T-N-**	**n**	**A-T+N-**	**P**	**P$**
Age (year)	105	76.7 (5.3)	104	78.0 (5.8)	0.1081	0.0598	71	76.5 (4.9)	6	74.0 (3.1)	0.1146	0.2670
Men/Women	105	40.00%	104	41.3%	0.9543	0.6907	71	42.3%	6	33.3%	1.0000	0.8751
e4/total genotyped	105	27.6%	104	45.2%	0.0125	0.0044	71	11.3%	6	50.0%	0.0459	0.0121
Hippocampal volume (R+L) (cm3)	88	4.69 (1.09)	90	4.44 (1.08)	0.1277	0.2611	60	4.72 (1.21)	6	5.38 (1.01)	0.1782	0.5884
MMSE	101	27.0 (25.0-29.0)	102	26.0 (25.0-28.0)	0.0096	0.0638	70	28.0 (26.0-29.0)	5	28.0 (24.0-29.0)	0.9400	0.7675
MMSE/year	98	-0.30 (-1.42-0.17)	93	-0.94 (-3.23--0.10)	0.0018	0.0199	66	-0.33 (-1.18-0.07)	6	-0.14 (-0.48-0.10)	0.3978	0.6189
Conversion to AD	105	17.1%	104	48.1%	< 0.0001	< 0.0001	71	11.3%	6	0.0%	0.8635	0.3391
Aβ38 (pg/mL)	96	1983 (795)	100	2683 (959)	< 0.0001	< 0.0001	66	2051 (795)	6	2231 (1026)	0.6920	0.9859
Aβ40 (pg/mL)	105	6492 (1725)	104	8426 (2203)	< 0.0001	< 0.0001	71	7224 (1677)	6	6664 (1682)	0.4633	0.3548
Aβ42 (pg/mL)	105	850 (506-1136)	104	577 (395-860)	0.0035	0.1108	71	1105 (275)	6	765 (213)	0.0092	0.0038
Aβ42/40	105	0.139 (0.089-0.159)	104	0.072 (0.051-0.097)	< 0.0001	< 0.0001	71	0.156 (0.145-0.164)	6	0.110 (0.106-0.112)	0.0007	< 0.0001
sAPPα (pg/mL)	96	30.9 (25.1-36.2)	100	34.7 (28.2-42.2)	0.0019	0.0021	66	31.4 (25.7-36.9)	6	30.8 (24.8-37.6)	0.8705	0.2991
sAPPβ (pg/mL)	96	39.5 (32.1-48.3)	100	47.9 (35.4-56.9)	0.0028	0.0012	66	39.8 (33.3-50.2)	6	40.6 (31.6-49.4)	0.8625	0.2772
Tau (pg/mL)	105	278 (218-358)	104	592 (462-750)	< 0.0001	< 0.0001	71	274 (212-328)	6	315 (287-344)	0.0982	0.0547
pTau181 (pg/mL)	105	48.0 (38.0-53.0)	104	79.5 (68.0-101.0)	< 0.0001	< 0.0001	71	46.7 (10.6)	6	53.7 (6.7)	0.0532	0.0297
pTau217 (pg/mL)	105	138.2 (96.7-264.7)	104	563.4 (306.1-827.5)	< 0.0001	< 0.0001	71	111.8 (42.0)	6	334.4 (81.9)	0.0010	< 0.0001
BACE1 (pg/mL)	105	1453 (1137-1635)	104	1868 (1541-2362)	< 0.0001	< 0.0001	71	1534 (1260-1719)	6	1552 (1239-1710)	0.7829	0.7340
Ng (pg/mL)	105	262 (202-337)	104	532 (385-673)	< 0.0001	< 0.0001	71	281 (221-363)	6	316 (255-348)	0.5494	0.9698
Ng/BACE1	105	18.8 (15.8-24.2)	104	27.0 (21.4-31.5)	< 0.0001	< 0.0001	71	18.3 (15.6-24.5)	6	21.6 (3.6)	0.2962	0.7860
**Variable ADNI cohort**	**n**	**CSF_pTau181-**	**n**	**CSF_pTau181+**	**P**	**P$**	**n**	**A-T-N-**	**n**	**A-T+N-**	**P**	**P$**
Age (year)	359	71.4 (7.3)	153	74.6 (8.2)	0.0001	< 0.0001	210	69.4 (65.6-75.0)	20	72.3 (68.9-76.8)	0.1257	0.2375
Men/Women	359	46.9%	153	51.9%	0.3843	0.6182	210	47.3%	20	50.0%	1.0000	0.9500
e4/total genotyped	359	29.9%	153	54.3%	< 0.0001	< 0.0001	210	19.1%	20	22.2%	0.9976	0.6311
Hippocampal volume (R+L) (cm3)	359	7946 (7220-8493)	152	7438 (6600-8080)	< 0.0001	0.0001	210	8035 (927)	20	7471 (1021)	0.0263	0.0441
Aβ40 (pg/mL)	359	16477 (4926)	153	22083 (5555)	< 0.0001	< 0.0001	210	16637 (4568)	20	16387 (5792)	0.8527	0.7342
Aβ42 (pg/mL)	359	1130 (739-1650)	153	736 (561-1061)	< 0.0001	0.5148	210	1334 (1007-1722)	20	1268 (801-1708)	0.3128	0.8344
Aβ42/40	359	0.078 (0.050-0.092)	153	0.034 (0.029-0.044)	< 0.0001	< 0.0001	210	0.087 (0.074-0.095)	20	0.080 (0.067-0.096)	0.3708	0.6647
Tau (pg/mL)	359	204 (166-248)	153	396 (327-481)	< 0.0001	< 0.0001	210	186 (154-228)	20	202 (163-256)	0.3450	0.5344
pTau181 (pg/mL)	359	17.7 (13.6-22.0)	153	38.8 (30.8-49.5)	< 0.0001	< 0.0001	210	15.5 (12.4-19.8)	20	16.5 (13.6-22.1)	0.4288	0.6010

Abbreviations: *e4* apolipoprotein E4, Mini–Mental State Examination, *sd* standard deviation, *Ng* neurogranin, *BACE1* β-site APP cleaving enzyme 1

### Relationship between CSF biomarkers in the BALTAZAR cohort

**Fig. 2:**
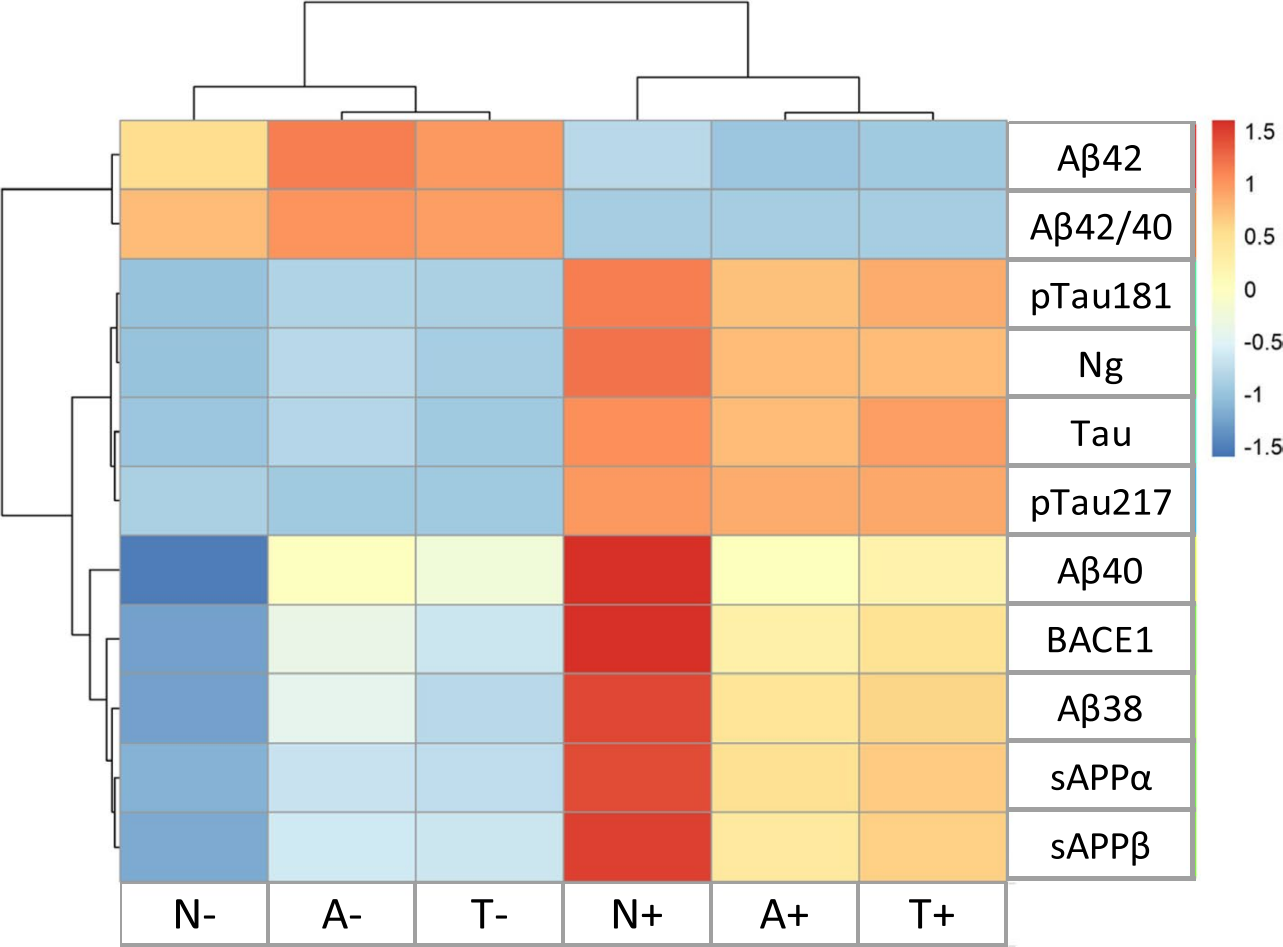
Unsupervised clustering of AT(N) status and CSF biomarkers in the BALTAZAR cohort. "In this representation, the individual biomarkers in each row are ordered based on their Euclidean distance, also illustrated by the dendrograms. Each column is ordered similarly and represents the A, T, N positive and negative situations (see supplementary Table 2). CSF biomarkers formed three distinct clusters. The first cluster grouped Aβ42 and Aβ42/40. The second cluster grouped Ng with Tau, pTau181, and pTau217. The third cluster grouped BACE1, Aβ40, Aβ38, sAPPα, and sAPPβ. ATN situations are also separated into positive and negative situations, with T and N closer together. The legend and the color gradient represent the variation of the biomarkers from low (blue) to high levels (red) in the different ATN subgroups

Table 3 revealed a high level of correlation (*r* > 0.6; *P* < 0.0001) of pTau217 with Aβ42 and Aβ42/40. Aβ38, Aβ40, sAPPα and sAPPβ are all correlated with each other (*r* > 0.4; *P* < 0.0001). BACE1 was correlated mainly with Aβ40 and this analyte with pTau181. Values of CSF Tau and pTau181 were highly corelated in both ADNI and BALTAZAR cohorts (*r* = 0.98 and *r *= 0.92 respectively; supplementary Fig. 3AB) and they both correlated partially with pTau217. The relationship between CSF biomarkers and AT(N) status is illustrated in Fig. 2 using an unsupervised clustering approach. Negative and positive status clustered apart. The N + status was clearly differential from the A + and T + that cluster together and similarly N- was separate from the A- and T- pairing. The CSF biomarkers form three distinct clusters. The first cluster contains Aβ42 and Aβ42/40 that reduce in AD stages. There were two clusters of markers that increase with the disease, one containing Ng, Tau, pTau181 and pTau217 and the other one with Aβ40 slightly separated from a group constituted of Aβ38, sAPPα, sAPPβ and BACE1.

**Table 3:** Correlation between CSF amyloid and tau biomarkers in the BALTAZAR cohort

	**Aβ40**	**Aβ42**	**Aβ42/40**	**sAPPα**	**sAPPβ**	**Tau**	**pTau181**	**pTau217**
Aβ40	/	0.44 (< 0.0001)	-0.11 (0.1132)	0.452 (< 0.0001)	0.508 (< 0.0001)	0.441 (< 0.0001)	0.556 (< 0.0001)	0.185 (0.0073)
Aβ42	0.44 (< 0.0001)	/	0.826 (< 0.0001)	0.14 (0.0509)	0.194 (0.0065)	-0.326 (< 0.0001)	-0.23 (0.0008)	-0.651 (< 0.0001)
Aβ42/40	-0.11 (0.1132)	0.826 (< 0.0001)	/	-0.122 (0.0875)	-0.099 (0.1682)	-0.614 (< 0.0001)	-0.575 (< 0.0001)	-0.811 (< 0.0001)
sAPPα	0.452 (< 0.0001)	0.14 (0.0509)	-0.122 (0.0875)	/	0.933 (< 0.0001)	0.317 (< 0.0001)	0.328 (< 0.0001)	0.221 (0.0019)
sAPPβ	0.508 (< 0.0001)	0.194 (0.0065)	-0.099 (0.1682)	0.933 (< 0.0001)	/	0.321 (< 0.0001)	0.317 (< 0.0001)	0.193 (0.0067)
Tau	0.441 (< 0.0001)	-0.326 (< 0.0001)	-0.614 (< 0.0001)	0.317 (< 0.0001)	0.321 (< 0.0001)	/	0.927 (< 0.0001)	0.781 (< 0.0001)
pTau181	0.556 (< 0.0001)	-0.23 (0.0008)	-0.575 (< 0.0001)	0.328 (< 0.0001)	0.317 (< 0.0001)	0.927 (< 0.0001)	/	0.748 (< 0.0001)
pTau217	0.185 (0.0073)	-0.651 (< 0.0001)	-0.811 (< 0.0001)	0.221 (0.0019)	0.193 (0.0067)	0.781 (< 0.0001)	0.748 (< 0.0001)	/

Correlation table between CSF biomarkers in the BALTAZAR cohort. The Spearman rank correlation coefficient is indicated, with *P* values in parentheses

## Discussion

One of the challenges to understanding the Alzheimer’s Disease (AD) continuum is to link biomarker profiles with the pathophysiology of the disease. To explore this relationship, we used the NIA-AA AT(N) framework [1] in the BALTAZAR prospective cohort [25] composed of MCI participants and in the ADNI cohort. Amyloid status A + was defined in the BALTAZAR cohort by the CSF Aβ42/40 ratio and in ADNI by amyloid PET. CSF total Tau was used to define neurodegeneration status N + in both cohorts.

Importantly, we realized that defining T + using CSF pTau181 was misleading, as CSF pTau181 corelates with CSF Tau (*r* > 0.9) (Supplementary Fig. 1) that is used to determined N + . Furthermore, CSF pTau181 does not correlate well with tau PET used to determined T + in the ADNI cohort. In our study, the use of pTau181 for stratification gave very similar results to the use of total tau which determines N + (Supplementary Tables 3 and 5, Supplementary Fig. 3). This observation raises concerns about the conclusion of numerus studies using CSF pTau181 to determine T + , including very recent ones using proteomics [16, 38, 43–47]. To define T + in BALTAZAR we rather used CSF pTau217 which correlates with tau PET [48, 49]. As expected, A + is associated with a significant decrease in Aβ42 in CSF. This goes hand in hand with the formation of Aβ oligomers and their aggregation in the brain parenchyma, leading to a reduction in soluble Aβ in CSF.

ApoE4, by reducing the clearance of Aβ or stimulating its production [50], strongly favours A + and it is not surprising that its prevalence is therefore high in this group. Tau proteins increased in A + patients, with pTau217 in BALTAZAR having the highest fold change and correlation with Aβ42/40. This is coherent with this biomarker being the best predictor of A + [51].

When we compare A-T-N- and A + T-N- cognition decline was not significant reminding therefore of cognitive unimpaired population that are at risk for AD. In this isolated A + situation, we noticed a decrease in BACE1, which is present in the presynaptic membrane, and Ng, which is predominantly localized post-synaptically and plays a role in long-term potentiation and learning. This result recalls a previous study [37]. The origin of this decrease could be related to Aβ-induced synaptic depression [52], feedback enzymatic inhibition [53], or alteration of synaptic structures [54]. Both biomarkers have similar expression patterns, with Ng showing a much stronger increase in the presence of neurodegeneration. The BACE1/Ng ratio, therefore, increases only in the later A + T + N + stage (Table 2), which is also associated with significant cognitive decline. Based on this observation, our interpretation is that the BACE1/Ng ratio, identified as an excellent biomarker of cognitive decline [18], is more a conjunctural construction than an association with pathophysiological significance, similar to the Aβ42/40 ratio. It will be interesting in future studies to examine other synaptic biomarkers [55], such as synaptosome-associated protein 25 (SNAP-25), growth-associated protein 43 (GAP-43), vesicle-associated membrane protein 2 (VAMP2) or neuronal pentraxin 2 (NPTX2), to see whether they show comparable variations as a function of ATN status.

The Alzheimer's disease continuum, notably modelled by Jack et al. [11], suggests that the natural evolution of the disease starts with A + followed by T + and eventually N + . Comparison of the whole T- and T + population revealed important differences in CSF biomarkers that corresponded well with those of AD: amyloidosis, increase in Tau isoforms, as well as in BACE1 and Ng, as already reported [56, 57]. However, if we focus on isolated T + (A-T + N-), we do not observe these modifications, which confirms that this profile is related to SNAP and does not belong to the AD continuum [16, 58, 59]. Taken together this suggest that T + only takes on its full pathological dimension when associated with A + . We however noticed that T + is associated with a small increase in BACE1 which could result from a direct activation mechanism by a truncated form of the Tau protein [60]. This truncated tau (1–368), generated by δ-secretase, which has also APP for substrate, results in BACE1 upregulation and Aβ production through binding to the transcription factor STAT1.

The addition of N + to A + T + represents the last step in Jack’s model and it is associated with all the hallmarks of AD (amyloid, tau, cognitive decline, increased Ng). However, contrary to the classic model we find a surprising association between N + and a coordinated increase in of BACE1, Aβ38, Aβ40, sAPPα and sAPPβ. The mechanism might be indirect, as BACE1 is regulated by oxidative stress, inflammation, insulin and interferon signaling and the receptor for advanced glycation end products [53, 61]. These factors and situations have been associated with tauopathy and could therefore account for the increased levels of BACE1, and subsequently, its metabolic products (Aβ38, Aβ40, sAPPβ). However, this does not explain the increase in sAPPα. Neurodegeneration itself could be another driving factor, as evidenced by the rise in total tau CSF levels and Ng levels (indicative of synaptic injury), which may be triggered by the activation of the injurious p75 neurotrophin receptor (p75) [62]. A recent study suggests that the increase of various CSF proteins including Aβ40, could result from altered CSF dynamics [47] an interesting hypotheses that would need further investigation. The authors also suggest that CSF protein concentration should be normalized with interindividual Aβ40 levels. However, in our dataset, this would have altered our N + population and modify the AT(N) classification, making our analysis inconsistent with previous studies.

One limitation of our study is that it is observational and limited to the concentration of biomarkers in CSF. It does not include anatomopathological investigations or ex vivo experimental approaches linking amyloid and tau pathologies. The observations are nevertheless supported by the analysis of two independent cohorts and PET imaging. There is a risk of circular thinking in the BALTAZAR cohort since we used CSF biomarkers for classification and analyzed the variation of CSF biomarkers. However, we were careful not to interpret the variation of the biomarkers used for each classification. Additionally, the fact that we reached similar conclusions using imaging biomarkers in the ANDI cohort adds to the robustness of our findings.

## Conclusions

Finally, an interesting illustration of the relationship between AT(N) and CSF biomarkers is provided by the unsupervised hierarchical clustering in Fig. 2. This representation shows, without a priori knowledge, that neurodegeneration augments amyloid constituents, with the exception of Aβ42 whose level decreases earlier along with amyloidopathy. As illustrated in the diagram of AD pathogenic events (Supplementary Fig. 4), our study thus clarifies the relationship between AT(N) profiles and AD pathophysiology. Our main finding is that CSF pTau181 is an indicator of N + rather than T + , and that N + is also associated with elevated levels of cerebrospinal fluid BACE1 protein and beta-amyloid peptides. This increase may potentially fuel the amyloid cascade in a positive feedback loop. Overall, our data provide further insights into understanding the interconnected pathological processes of amyloid, tau, and neurodegeneration underlying AD.

## Supplementary Information

Supplementary Material 1.

## Data Availability

Data and informed consent form are available upon request after publication (APHP, Paris). Requests will be considered by each study investigator, based on the information provided by the requester, regarding the study and analysis plan. If the use is appropriate, a data sharing agreement will be put in place before distributing a fully de-identified version of the dataset, including the data dictionary used for analysis with individual participant data.
