# Supplementary material for: Clarifying the association of CSF Aβ, tau, BACE1, and neurogranin with AT(N) stages in Alzheimer disease
Source: Mol Neurodegener. 2024 Oct 8;19:66. doi: 10.1186/s13024-024-00755-3 (PMC11460012; doi:10.1186/s13024-024-00755-3)
Supplement: Supplementary file 1 — Supplementary Material 1. [file 13024_2024_755_MOESM1_ESM.pdf]

Supplementary Table 1

	BALTAZAR		ADNI	
ATN status	**n**	**%**	**n**	**%**
A+T-N-	26	12.4%	59	11.5%
A-T+N-	10	4.8%	20	3.9%
A-T-N+	3	1.4%	33	6.4%
A+T+N-	4	1.9%	54	10.5%
A+T-N+	9	4.3%	30	5.9%
A-T+N+	14	6.7%	9	1.8%
A+T+N+	76	36.4%	97	18.9%
A-T-N-	67	32.1%	210	41.0%
ATN status	n	%	**n**	**%**
A+	115	55.0%	240	46.9%
T+	104	49.8%	180	35.2%
N+	102	48.8%	169	33.0%

Distribution of the participants between the different ATN status

Supplementary Table 2

ATN status	A+	N+	T+
**A-T-N-**			
**A+T-N-**	x		
**A+T+N-**	x		x
**A+T+N+**	x	x	x
**A+T-N+**	x	x	
**A-T+N-**			x
**A-T-N+**		x	
**A-T+N+**		x	x

Distribution of the ATN status

Supplementary Table 3

Baltazar cohot	n	A- (CSF)	n	A+ (CSF)	P	P$	n	T- (CSF)	n	T+ (CSF)	P	P$	n	N- (CSF)	n	N+ (CSF)	P	P$
Age (year)	115	*76.7 (5.2)*	94	*77.9 (5.9)*	0.0964	/	97	*77.0 (5.3)*	112	*77.7 (5.9)*	0.3671	/	107	*77.1 (5.2)*	102	77.6 (6.1)	0.5579	/
Men/Women	115	45.7%	94	36.5%	0.2267	/	97	43.3%	112	38.4%	0.5627	/	107	39.3%	102	42.2%	0.7745	/
e4/total genotyped	115	16,00%	94	53,00%	<0.0001	/	97	15.5%	112	54.5%	<0.0001	/	107	25.2%	102	48,00%	0.001	/
Hippocampal vol	98	*4.62 (1.24)*	80	*4.52 (0.95)*	0.5336	0.9774	80	*4.67 (1.21)*	98	*4.48 (0.97)*	0.261	0.4989	91	*4.73 (1.11)*	87	*4.39 (1.04)*	0.0383	0.046
MMSE	112	27.0 (26.0-29.0)	91	26.0 (24.0-28.0)	0.0001	0.0006	94	27.0 (26.0-29.0)	109	26.0 (24.0-28.0)	0.0008	0.0145	103	27.0 (25.0-29.0)	100	26.0 (25.0-28.0)	0.0095	0.0979
MMSE/year	104	-0.46 (-1.43-0.07)	87	-0.89 (-3.26-0.04)	0.1271	0.1379	89	-0.36 (-1.38-0.07)	102	-0.89 (-3.77-0.00)	0.0225	0.0209	100	-0.33 (-1.25-0.12)	91	-1.00 (-3.93--0.00)	0.0025	0.0095
Aβ38 (pg/mL)	107	2160 (1674-2947)	89	2389 (1794-3033)	0.2223	0.7512	92	2071 (1577-2915)	104	2435 (1873-3041)	0.0442	0.443	100	1942 (1463-2442)	96	2666 (2126-3284)	<0.0001	<0.0001
Aβ40 (pg/mL)	115	*7444 (1832)*	94	*7463 (2465)*	0.9466	0.6186	97	*7350 (2142)*	112	*7545 (2251)*	0.5226	0.8232	107	*6798 (1884)*	102	*8144 (2299)*	<0.0001	<0.0001
Aβ42 (pg/mL)	115	1129 (897-1293)	94	494 (353-591)	<0.0001	<0.0001	97	1082 (844-1264)	112	502 (356-633)	<0.0001	<0.0001	107	945 (588-1158)	102	548 (378-696)	<0.0001	<0.0001
Aβ42/40	115	0.156 (0.134-0.163)	94	0.070 (0.052-0.083)	***	***	97	0.154 (0.122-0.163)	112	0.069 (0.052-0.084)	<0.0001	<0.0001	107	0.145 (0.095-0.162)	102	0.070 (0.051-0.087)	<0.0001	<0.0001
sAPPα (pg/mL)	107	31.4 (25.8-36.8)	89	33.9 (27.5-41.2)	0.1077	0.5858	92	31.2 (25.6-36.4)	104	34.2 (27.7-41.4)	0.0303	0.3085	100	30.4 (24.9-35.1)	96	35.9 (28.6-42.7)	0.0003	0.0019
sAPPβ (pg/mL)	107	40.3 (34.0-50.6)	89	44.5 (34.5-55.9)	0.2162	0.6457	92	40.2 (33.4-50.3)	104	45.5 (35.1-55.8)	0.0791	0.5017	100	37.8 (32.6-46.9)	96	49.2 (36.8-57.2)	0.0001	0.0006
Tau (pg/mL)	115	302 (226-381)	94	556 (391-730)	<0.0001	<0.0001	97	283 (218-375)	112	587 (408-740)	<0.0001	<0.0001	107	279 (218-348)	102	603 (503-760)	***	***
pTau181 (pg/mL)	115	50.5 (40.2-59.5)	94	74.0 (55.5-98.0)	<0.0001	<0.0001	97	50.0 (38.0-58.0)	112	75.5 (57.8-99.0)	<0.0001	<0.0001	107	48.0 (38.5-54.5)	102	80.0 (68.0-103.0)	<0.0001	<0.0001
pTau217 (pg/mL)	115	120.4 (84.8-166.7)	94	540.7 (328.7-790.2)	<0.0001	<0.0001	97	120.7 (84.8-166.1)	112	548.4 (356.4-798.5)	***	***	107	133.3 (96.1-219.4)	102	567.8 (338.8-822.5)	<0.0001	<0.0001
BACE1 (pg/mL)	115	1566 (1272-1776)	94	1639 (1224-2094)	0.2638	0.2356	97	1534 (1234-1781)	112	1666 (1304-2093)	0.0222	0.0194	107	1458 (1148-1686)	102	1804 (1472-2400)	<0.0001	<0.0001
Ng (pg/mL)	115	297 (236-380)	94	477 (294-608)	<0.0001	0.0001	97	285 (218-375)	112	478 (316-615)	<0.0001	<0.0001	107	273 (208-363)	102	531 (378-677)	<0.0001	<0.0001
Ng/BACE1	115	20.1 (16.7-25.0)	94	25.0 (19.8-31.3)	<0.0001	0.0016	97	19.2 (16.0-25.0)	112	25.2 (20.6-31.2)	<0.0001	0.0003	107	19.5 (15.8-24.3)	102	27.5 (21.1-31.8)	<0.0001	<0.0001
**ADNI cohort**	**n**	**A- (PET)**	**n**	**A+ (PET)**	**P**	**P$**	**n**	**T- (PET)**	**n**	**T+ (PET)**	**P**	**P$**	**n**	**N- (CSF)**	**n**	**N+ (CSF)**	**P**	**P$**
Age (year)	272	69.9 (65.9-75.2)	240	74.6 (69.3-79.7)	<0.0001	/	332	71.0 (66.3-76.7)	180	73.8 (68.7-79.1)	0.0077	/	343	*71.4 (7.4)*	169	*74.4 (8.1)*	0.0002	/
Men/Women	272	48.2%	240	48.5%	1	/	332	48.7%	180	47.6%	0.9144	/	343	47.4%	169	50.4%	0.6299	/
e4/total genotyped	272	20.4%	240	56.9%	<0.0001	/	332	28.7%	180	53.7%	<0.0001	/	343	31.4%	169	48.9%	0.0005	/
Hippocampal vol	271	8009 (7378-8588)	240	7510 (6651-8183)	<0.0001	0.0002	331	7988 (935)	180	7148 (1119)	<0.0001	<0.0001	343	7896 (7224-8480)	168	7526 (6581-8210)	<0.0001	0.0003
Aβ40 (pg/mL)	272	*18108 (5798)*	240	*18202 (5654)*	0.8533	0.3429	332	17570 (14162-21523)	180	18205 (14578-22425)	0.3408	0.8122	343	*16065 (4654)*	169	*22388 (5356)*	<0.0001	<0.0001
Aβ42 (pg/mL)	272	1404 (1041-1892)	240	689 (477-860)	<0.0001	<0.0001	332	1210 (826-1702)	180	695 (480-888)	<0.0001	<0.0001	343	1098 (722-1612)	169	758 (607-1285)	0.0009	0.091
Aβ42/40	272	0.087 (0.073-0.096)	240	0.037 (0.031-0.047)	<0.0001	<0.0001	332	0.080 (0.053-0.093)	180	0.037 (0.030-0.049)	<0.0001	<0.0001	343	0.077 (0.049-0.092)	169	0.036 (0.030-0.054)	<0.0001	<0.0001
Tau (pg/mL)	272	204 (161-257)	240	295 (236-427)	<0.0001	<0.0001	332	213 (169-266)	180	313 (248-439)	<0.0001	<0.0001	343	201 (163-243)	169	376 (320-467)	***	***
pTau181 (pg/mL)	272	17.3 (13.2-22.0)	240	28.0 (21.2-41.8)	<0.0001	<0.0001	332	18.7 (13.7-23.4)	180	30.3 (23.0-45.7)	<0.0001	<0.0001	343	17.5 (13.6-21.7)	169	36.1 (30.0-49.2)	<0.0001	<0.0001

Demographics and CSF biomarker values in the number (n) of participants in A+, T+ and N+ populations. Comparison between global A-/A+, T-/T+ and N-/N+ profiles. Categorical variables were reported as percentage (%), continuous variables as mean (standard deviation) or as median (25-75 percentile) after testing for normal distribution using the Shapiro-Wilk test. This explains why, for the same subgroup of patients, a variable could be represented either as the mean (standard deviation), italicized, or as the median (25th-75th percentile). P values of comparison made by $\chi 2$ test, T-test, or Wilcoxson test. P$ values of comparison with linear regression adjusted for age, sex, and the presence of the APOE ε4 allele. *** indicates the biomarker used for stratification.

Abbreviations: e4: apolipoprotein E4; Mini–Mental State Examination; sd, standard deviation; Ng, neurogranin; BACE1, β-site APP cleaving enzyme 1.

Supplementary Table 4

ADNI cohort	n	A-	n	A+	P	n	N-	n	N+	P
sAPPβ (pM)	114	4001 (3128-5157)	263	3839 (3107-4860)	0.1420	183	3457 (2596-4615)	194	4409 (3530-52194)	<0.0001
BACE1 (pM)	114	45.5 (36.0-61.0)	263	43.0 (30.2-57.0)	0.0244	183	38.0 (30.0-48.0)	194	51.0 (36.0-56.0)	<0.0001

Comparison of values in the number (n) of participants within the different status. In this ADNI subcohort the A+ status was defined based on CSF Aβ42 value and the N+ on the CSF total tau value. Variables were reported as median (25-75 percentile). P values of comparison made with Wilcoxson test. Abbreviations: BACE1, β-site APP cleaving enzyme 1.

Supplementary Table 5

Baltazar cohot	n	CSF_pTau181-	n	CSF_pTau181+	P	P$
Age (year)	105	*76.7 (5.3)*	104	*78.0 (5.8)*	0.1081	0.0598
Men/Women	105	40,00%	104	41.3%	0.9543	0.6907
e4/total genotyped	105	27.6%	104	45.2%	0.0125	0.0044
Hippocampal vol	88	*4.69 (1.09)*	90	*4.44 (1.08)*	0.1277	0.2611
MMSE	101	27.0 (25.0-29.0)	102	26.0 (25.0-28.0)	0.0096	0.0638
MMSE/year	98	-0.30 (-1.42-0.17)	93	-0.94 (-3.23--0.10)	0.0018	0.0199
Aβ38 (pg/mL)	96	*1983 (795)*	100	*2683 (959)*	<0.0001	<0.0001
Aβ40 (pg/mL)	105	*6492 (1725)*	104	*8426 (2203)*	<0.0001	<0.0001
Aβ42 (pg/mL)	105	850 (506-1136)	104	577 (395-860)	0.0035	0.1108
Aβ42/40	105	0.139 (0.089-0.159)	104	0.072 (0.051-0.097)	<0.0001	<0.0001
sAPPα (pg/mL)	96	30.9 (25.1-36.2)	100	34.7 (28.2-42.2)	0.0019	0.0021
sAPPβ (pg/mL)	96	39.5 (32.1-48.3)	100	47.9 (35.4-56.9)	0.0028	0.0012
Tau (pg/mL)	105	278 (218-358)	104	592 (462-750)	<0.0001	<0.0001
pTau181 (pg/mL)	105	48.0 (38.0-53.0)	104	79.5 (68.0-101.0)	***	***
pTau217 (pg/mL)	105	138.2 (96.7-264.7)	104	563.4 (306.1-827.5)	<0.0001	<0.0001
BACE1 (pg/mL)	105	1453 (1137-1635)	104	1868 (1541-2362)	<0.0001	<0.0001
Ng (pg/mL)	105	262 (202-337)	104	532 (385-673)	<0.0001	<0.0001
Ng/BACE1	105	18.8 (15.8-24.2)	104	27.0 (21.4-31.5)	<0.0001	<0.0001
**ADNI cohort**	**n**	**CSF_pTau181-**	**n**	**CSF_pTau181+**	**P**	**P$**
Age (year)	359	*71.4 (7.3)*	153	*74.6 (8.2)*	0.0001	<0.0001
Men/Women	359	46.9%	153	51.9%	0.3843	0.6182
e4/total genotyped	359	29.9%	153	54.3%	<0.0001	<0.0001
Hippocampal vol	359	7946 (7220-8493)	152	7438 (6600-8080)	<0.0001	0.0001
Aβ40 (pg/mL)	359	*16477 (4926)*	153	*22083 (5555)*	<0.0001	<0.0001
Aβ42 (pg/mL)	359	1130 (739-1650)	153	736 (561-1061)	<0.0001	0.5148
Aβ42/40	359	0.078 (0.050-0.092)	153	0.034 (0.029-0.044)	<0.0001	<0.0001
Tau (pg/mL)	359	204 (166-248)	153	396 (327-481)	<0.0001	<0.0001
pTau181 (pg/mL)	359	17.7 (13.6-22.0)	153	38.8 (30.8-49.5)	***	***

Comparison of values in the number (n) of participants within the different status. Categorical variables were reported as percentage (%), continuous variables as mean (standard deviation) or as median (25-75 percentile) after testing for normal distribution using the Shapiro-Wilk test. This explains why, for the same subgroup of patients, a variable could be represented either as the mean (standard deviation), italicized, or as the median (25th-75th percentile). P values of comparison made by $\chi 2$ test, T-test, or Wilcoxson test. P$ values of comparison with linear regression adjusted for age, sex, and the presence of the APOE ε4 allele. *** indicates the biomarker used for stratification.

Abbreviations: e4: apolipoprotein E4; Mini–Mental State Examination; sd, standard deviation; Ng, neurogranin; BACE1, β-site APP cleaving enzyme 1.

Supplementary Figure 1

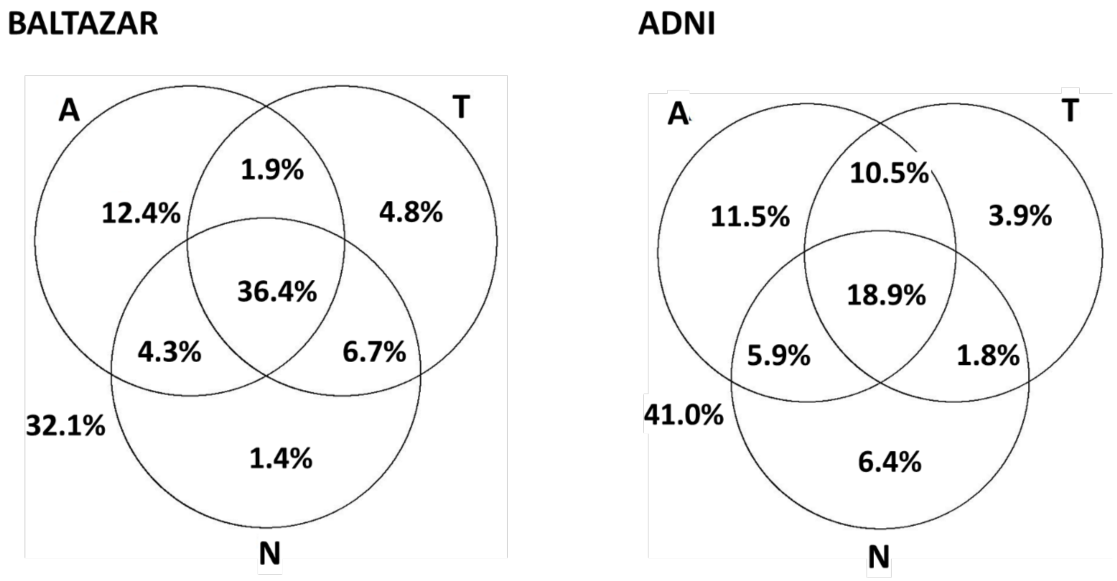

Venn diagram of the ATN status in the BALTAZAR and the ADNI population.
A, T and N populations correspond each to one circle, 32.1% participant of BALTAZAR and 41.0% of ADNI are A-T-N- (represented outside the circles).

Supplementary Figure 2

Violin plot distribution of Aβ40 and Aβ42 CSF levels in the BALTAZAR (A, B) and the ADNI (I, J) cohorts, stratified by AT(N) classification showing median and quartiles. Aβ40 levels were statistically different between the N- and N+ groups. Aβ38 levels (C) showed an statistically significant increase with the presence of N+. Aβ42 levels were statistically decreased in A+, T+ and N+ compared to the respective A-, T- and N- populations. We also showed the variation of Aβ42/40 ratio (D) which is used for A+ stratification. sAPPs distribution (E, F) are similar to that of Aβ40. Ng (G), as a synaptic biomarker increased in A+, T+ and N+. BACE1 (H) increased a little in T+ and more in N+. P values of Wilcoxson test <0.001 are indicated with ***, <0.01 with ** and <0.05, with *.

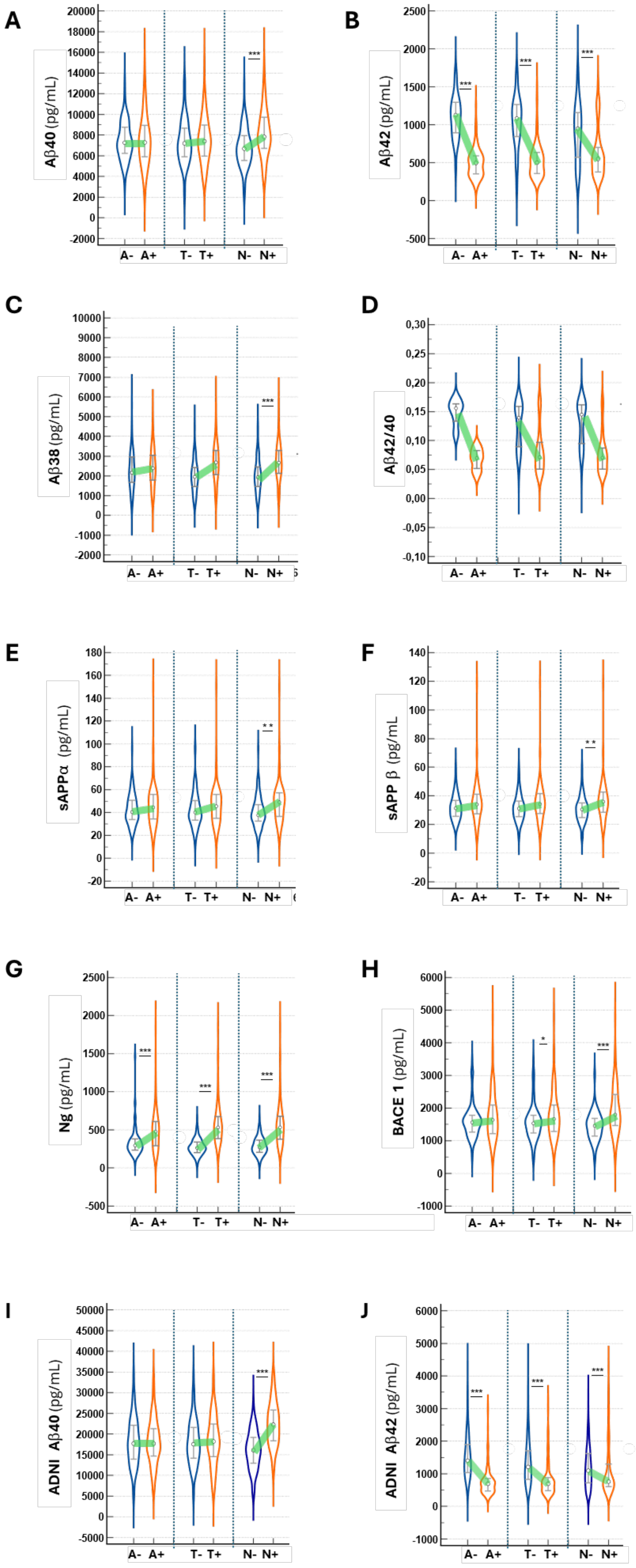

Supplementary Figure 3

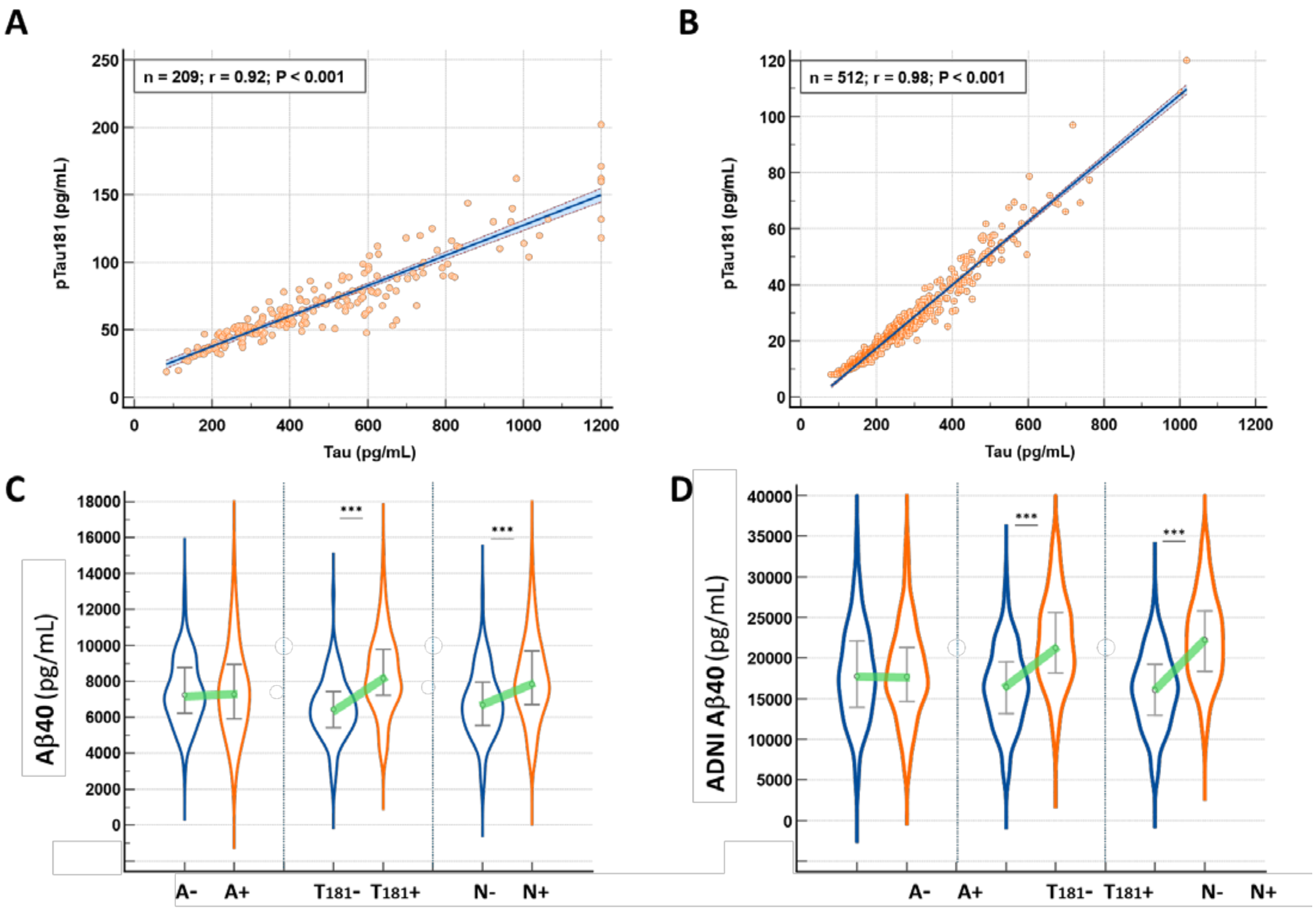

Corelation plot of CSF Tau and pTau181 in the BALTAZAR (A) and the ADNI cohort (B). Distribution of Aβ40 CSF levels illustrated using violin plot and median/25-75th percentile in the BALTAZAR (C) and the ADNI (D) cohorts in the population stratified by the presence of amyloidosis (A-/+), using pTau181 (T181-/+) or Tau (N-/+). classification illustrated using violin plot and median/25-75th percentile indication. P values of Wilcoxson test <0.001 are indicated with ***.

Supplementary Figure 4

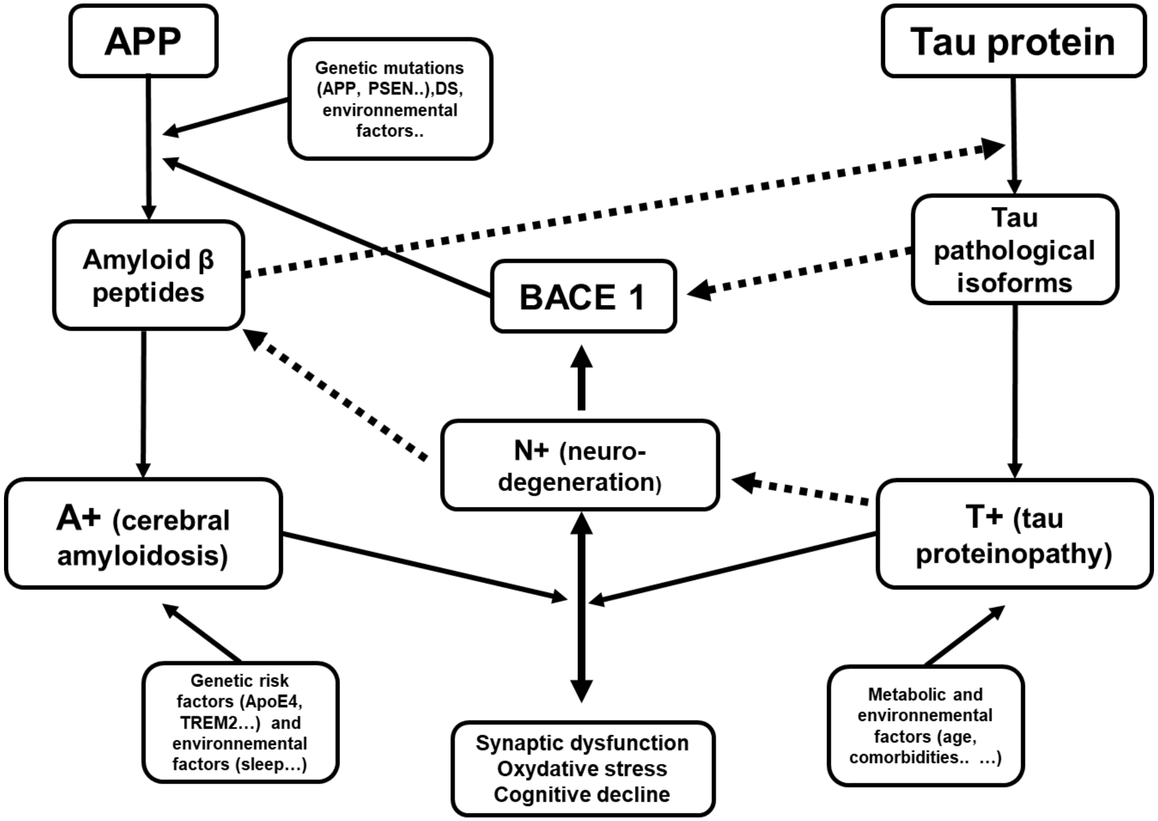

Molecular pathways in Alzheimer's Disease involving synergic pathological interplay between amyloid and tau.
APP protein is metabolized by secretases, including BACE1, producing various amyloid Aβ and sAPP peptides of different lengths. These processes are influenced by genetic mutations linked to familial AD, such as mutations in APP and PSEN1/2 genes. Cerebral amyloidosis results from the metabolic dysregulation and aggregation of pathogenic Aβ peptides. This transition is further influenced by genetic variants like ApoE and TREM2. Pathological tau modifications, triggered notably by Aβ peptides, increase BACE1 activity. In association with amyloidosis, this cascade leads to neurodegeneration. As our study detects, neurodegeneration is central to this scheme, driving further increases in BACE1 and Aβ peptides.
Abbreviations: APP, Amyloid precursor protein; BACE1, β-site APP cleaving enzyme 1; DS, Down's syndrome; PSEN. presenilin.
